# House dust mite exposure enhances immune responses to ovalbumin-induced intestinal allergy

**DOI:** 10.1038/s41598-022-09196-8

**Published:** 2022-03-25

**Authors:** Jianli Lin, Desheng Chen, Lvxin Guan, Kexin Chang, Dan Li, Baoqing Sun, Pingchang Yang, Zhigang Liu

**Affiliations:** 1 State Key Laboratory of Respiratory Disease Allergy Division at Shenzhen University, Institute of Allergy & Immunology, Shenzhen University School of Medicine, Room 509 of A7 Bldgn, 1066 Xueyuan Blvd, Shenzhen, 518055 China; 2 State Key Laboratory of Respiratory Disease, Department of Allergy and Clinical Immunology, National Clinical Center for Respiratory Diseases, Guangzhou Institute of Respiratory Diseases, First Affiliated Hospital, Guangzhou Medical University, 151 Yanjiangxi Road, Guangzhou, 510120 Guangdong Province China; 3 Department of General Surgery, Shanghai General Hospital, Shanghai Jiaotong University School of Medicine, Wujin road 85, Shanghai, 200080 China

**Keywords:** Pathogens, Antibodies

## Abstract

House dust mites (HDM) are one of the important factors of airway allergic diseases, HDM allergens can be detected in the human gut mucosa, which induces local inflammation and increases intestinal epithelial permeability. This study tests a hypothesis that HDM contribute to the development of OVA (ovalbumin)-induced intestinal allergy. The serum levels of IgE against HDM in patients with food allergy were detected with UniCAP100 (Pharmacia, Uppsala, Sweden); a mouse model of food allergy was developed with OVA and HDM as the specific antigens. Compared to healthy controls, patients with food allergy have higher levels of serum HDM-specific IgE. Compared to food allergy alone groups, the levels of HDM-specific IgE in patients with food allergy and asthma or allergic rhinitis were significantly higher. In mouse models, we found that HDM/OVA induced allergy-like symptoms, lower body temperature, and lower body weight. The levels of IgE, IgG1, mMCP-1 (mouse mast cell protease-1), IL-4 and IL-5 in the HDM and HDM + CT (cholera toxin) groups were higher than the control groups, and the levels of IgE, IgG1, IL-4 and IL-5 in the HDM, OVA and HDM + OVA groups were higher than the control groups. The pathological changes of intestinal tissues in the HDM and HDM + CT/the HDM, OVA and HDM + OVA groups were more severe, more eosinophil infiltration than the control groups. Moreover, exposure to HDM induced intestinal barrier dysfunction, and facilitated the development of intestinal allergy in mice. In conclusion, HDM exposure enhances immune responses to OVA-induced food allergy.

## Introduction

Food allergy is estimated to affect about 5% adults and 8% young children; the prevalence of food allergy has increased significantly in the recent decades^1,2^. Food allergy has become a worldwide health problem, which burdens patients and families by increasing expenses of healthcare and negatively affects the quality of life.

There are 8 foods most commonly identified by the U.S. Food and Drug Administration (FDA), that include peanuts, tree nuts, soy, wheat, fish, shellfish, milk and eggs^3^. Food allergens usually get into the digestive tract straight through diet, and then, possibly, induce intestinal sensitization/allergy.

There is a high coincidence between food allergy and other allergic diseases, such as allergic asthma, allergic rhinitis and atopic dermatitis^4^. Asthmatic patients appear at significantly increased risk of severe allergic reactions induced by food allergens^5^. The cross-reactivity between aeroallergens and food allergens may induce food allergy in patients with airway allergy^6^. Especially, tropomyosin was involved in cross-reactivity between house dust mite (HDM) and shrimp, thus shrimp and HDM allergies usually occur in the same patient, and consequently, the frequency of HDM sensitization in shrimp allergic people is higher^7^.

There are many seasonal/outdoor aeroallergens, such as dust mites, pollen, cockroaches, fungi and animal feathers^8^. Nearly80% of asthmatic patients are sensitized to HDM, the predominant sources of aeroallergens^9^. In recent research, HDM allergens were detected in the human intestine, which induced local inflammation and increased intestinal epithelial permeability^10^.

Based on the information above, we hypothesize that HDM may be not only an aeroallergen, but also an important allergen in food allergy. In this study, the levels of specific IgE against HDM in patients with food allergy were analyzed, and an HDM/OVA-induced mouse model of food allergy was developed. We found that exposure to HDM increased the intestinal epithelial barrier permeability, and facilitated the development of intestinal allergy in mice. The data demonstrate that HDM not only is an aeroallergen, which induces airway allergy, but also enhances subsequent immune responses to OVA-induced food allergy.

## Materials and methods

### Human subjects

In a total of 825 food allergic patients’ serum were collected at the First Affiliated Hospital, Guangzhou Medical University (Guangzhou, China) during January 2015 to November 2016. The diagnosis of food allergy was conducted by the doctors of this hospital. The clinical features of human subjects are presented in Table 1. All subjects of allergic rhinitis alone or asthma alone met the following inclusion criteria: (1) Physician-diagnosed allergic rhinitis according to the Allergic Rhinitis and its Impact on Asthma guideline (ARIA); (2) Physician-diagnosed asthma according to the Global Initiative for Asthma guideline (GINA); (3) Healthy controls had no prior history of respiratory disease. (4) All subjects have no prior history of autoimmune disease. Subjects were excluded if they had (1) physician-diagnosed other respiratory diseases, including chronic obstructive pulmonary disease, bronchiectasis, pneumonia, or pulmonary tuberculosis; (2) Complicated with critical illness of other organs, coma, shock, or disturbance of consciousness; (3) Patients of pregnancy or breastfeeding. After comparing the age data between groups, the difference had no significance. This study has been approved by the Human Ethics Committee at Shenzhen University and Guangzhou Medical University. Informed consent was obtained from all subjects and if subjects are under 18, from a legal guardian. All experiments were performed in accordance with the relevant guidelines and regulations.

**Table 1:** Clinical features of human subjects.

	Food allergy (n = 825)	Healthy subjects (n = 25)
Sex M/F	528/297 (M-64%)	14/11 (M-56%)
Age mean; range	11.46 ± 0.6051 (1–87)	17.46 ± 4.761 (1–82)
Personal history of atopy	332 (40.24%)	0
Total IgE	447.7 ± 21.64**	109.8 ± 17.76
**Atopic comorbidities**		
Bronchcial asthma	223 (27.03%)	0
Allergic rhinitis	83 (10.06%)	0
Allergic dermatitis	26 (3.15%)	0
Positive sIgE to HDM	544 (65.94%)	20%
**Allergen distribution**		
Milk	511 (61.94%)	0
Egg	419 (50.79%)	0
Shrimp	223 (27.03%)	0
Crab	114 (13.82%)	0
Wheat	57 (6.91%)	0
Peanut	12 (1.45%)	0
Soy	12 (1.45%)	0
Fish	2 (0.24%)	0

### *Dermatophagoides pteronyssinus* culture and extracts preparation

*Dermatophagoides pteronyssinus* mites were cultured as reported previously^11^, dust mites were cultured at 25 °C with 70% relative humidity. Subsequently, mites were isolated from the medium using a modified heat-escape method and the dust mite purity was evaluated by checking mite morphology. Mite bodies were washed with PBS, weigh 2-g sample adding 1 ml lysate (9 M urea, 4% CHAPS, 60 mM DTT, 2% IPG buffer) and homogenized in liquid nitrogen, centrifuged at 15,000 rpm for 20 min under refrigeration. The supernatant was termed HDM extract.

### Mice

6 to 8 weeks old female BALB/c mice (weight: 18–20 g), obtained from the Guangdong Experimental Animal Center (Guangzhou, China), were maintained in specific pathogen-free conditions according to standard guidelines for the care and use of animals. The experimental procedures were approved by the Institutional Ethics Committee at Shenzhen University (Shenzhen, China). The study was carried out in compliance with the ARRIVE guidelines. Laboratory animal ethics committee, Shenzhen Research Institute, Hong Kong Polytechnic University: #161,201. The experiments were performed in accordance with the approved guidelines.

### Induction of experimental food allergy

As shown by Fig. 2A, 18 mice were randomly divided into 3 groups: HDM + cholera toxin (CT) group, HDM group and Control group. Mice were sensitized by intraperitoneal injection with PBS (Control group), HDM extract (1 mg/mouse) and CT (20 μg/mouse) (HDM + CT group), or HDM extract (1 mg/mouse) (HDM group) on day 0 and day 3, respectively. From day 5 on, challenge was performed every other day for 10 days, including that mice were challenged with PBS (Control group), HDM extract (1 mg/mouse) and CT (20 μg/mouse) (HDM + CT group), or HDM extract (1 mg/mouse) (HDM group) by intra-gastric (i.g) gavage. The body weight of each mouse was recorded every other day. An OVA food allergy followed by HDM exposure was also induced in Balb/c mice. Mice were exposed to HDM (1 mg, i. g)/PBS daily for one week. Combination of HDM exposure and food allergy was performed with the two protocols (Fig. 4A). Control mice receivedPBS injection alone and challenged with PBS alone.

### Enzyme-linked immunosorbent assay (ELISA)

The levels of specific IgE and IgG1 for HDM were determined by ELISA as described previously^12^. Briefly, the ELISA microtiter plates were coated with HDM with at 1 ug/well in 100 µl carbonate buffered solution (CBS, 15 mM Na_2_CO_3_ and 35 mM NaHCO_3_, pH9.5). After incubation (overnight, 4 °C), plates were washed 3 times with PBST (PBS containing 0.05% Tween 20), and blocked with 3% bovine serum albumin in PBS (3% BSA-PBS) (1 h, 37 °C). The serum (1:10 diluted with 3% BSA-PBS) or BSA (using as a negative control) were then added to each well and incubated (2 h, 37 °C). Subsequently, 100 µL of peroxidase-labeled goat anti-mouse IgE (1:2000) was added to each well. The plates were incubated (2 h, 37 °C). Following 3 washings, the reactions were developed with TMB (tetramethylbenzidine, 100uL/well) for 20 min and stopped by 50 µl 2 M H2SO4. The plates were read by an ELx808 absorbance microplate reader (BioTek, Shanghai, China) at 450 nm. The splenocytes culture supernatant levels cytokines IL-4 (Boster, Wuhan, China), IL-5 and IFN-γ (Sino Biological Inc, Beijing, China) were determined by ELISA with commercial reagent kits following the manufacturer’s instruction.

### Immuno-CAP 1000 system

Immuno-CAP 1000 system (Thermo Fisher Scientific Inc., USA) was used to detect allergen-specific IgE (sIgE) in the sera. Enzyme-linked fluorescence assay was performed according to the manufacturer’s instructions for sIgE detection. This protocol was performed by the First Affiliated Hospital, Guangzhou Medical University (Guangzhou, China). SIgE against HDM was evaluated in this study and the cut-off value of positive reactivity was 0.35 kU/L.

### Flow cytometry

Spleen cells were prepared according to Gunzer M, et al.’s report^13^. Splenocytes (2 × 10^6^/well) were labeled with CFSE (5,6-carboxyfluorescein diacetate, succinimidyl ester) in the dark, incubated with 50 μg/ml HDM or culture medium and 2 μl/ml cell stimulation cocktail (a cocktail of phorbol 12-myristate 13-acetate (PMA) and ionomycin, ebioscience) in 96-well plate (72 h, 37 °C). Following washing with PBS, the cells were stained with CD4-APC (1 h, room temperature). After washing, the cells were analyzed with a flow cytometer (BD Bioscience FACSCanto II). The data were analyzed with software Flowjo.

### Assessment of the intestinal permeability in vivo

This measure is based on the intestinal epithelial barrier permeability to 4,000-Da fluorescent-dextran (Sigma-Aldrich)^14^. 6-h-fasted mice were fed with fluorescein isothiocyanate (FITC)-dextran at 600 mg/kg body weight (125 mg/ml PBS). After 1 h, the mice were sacrificed. The blood was collected from the tip of the tail vein. The blood was centrifuged at 5000 rpm (3 min, 4 °C.) Plasma was diluted in an equal volume of PBS (pH 7.4) and the FITC-dextran concentrations in the plasma were determined with a fluorescence spectrophotometer at the excitation wavelength of 485 nm and the emission wavelength of 535 nm. Standard curves for calculating the FITC-dextran concentration in the plasma were obtained by diluting FITC-dextran in nontreated plasma diluted with PBS (1:2 [vol/vol]).

### Histology

Jejunum samples were fixed in 4% formalin overnight and embedded in paraffin. The tissues were cut into 4-µm thick sections and stained with hematoxylin and eosin (HE). The numbers of eosinophils and mononuclear cells in the jejunum tissues were counted under a light microscope. To avoid the observer bias, sections were coded; observers were not aware of the code.

### Statistics

Data are presented as mean ± SD. Difference between 2 groups was determined by Student *t* test or ANOVA if more than two groups. *P* < 0.05 was set as a significant criterion. All data were processed with GraphPad Prism software.

### Ethics approval

The ethics approval number from Laboratory animal ethics committee, Shenzhen Research Institute, Hong Kong Polytechnic University was #161,201, and 201,540 from Medical ethics committee, The First Affiliated Hospital, Guangzhou Medical University.

## Results

### Patients with food allergy have higher levels of HDM-specific IgE in the serum

**Figure 1:**
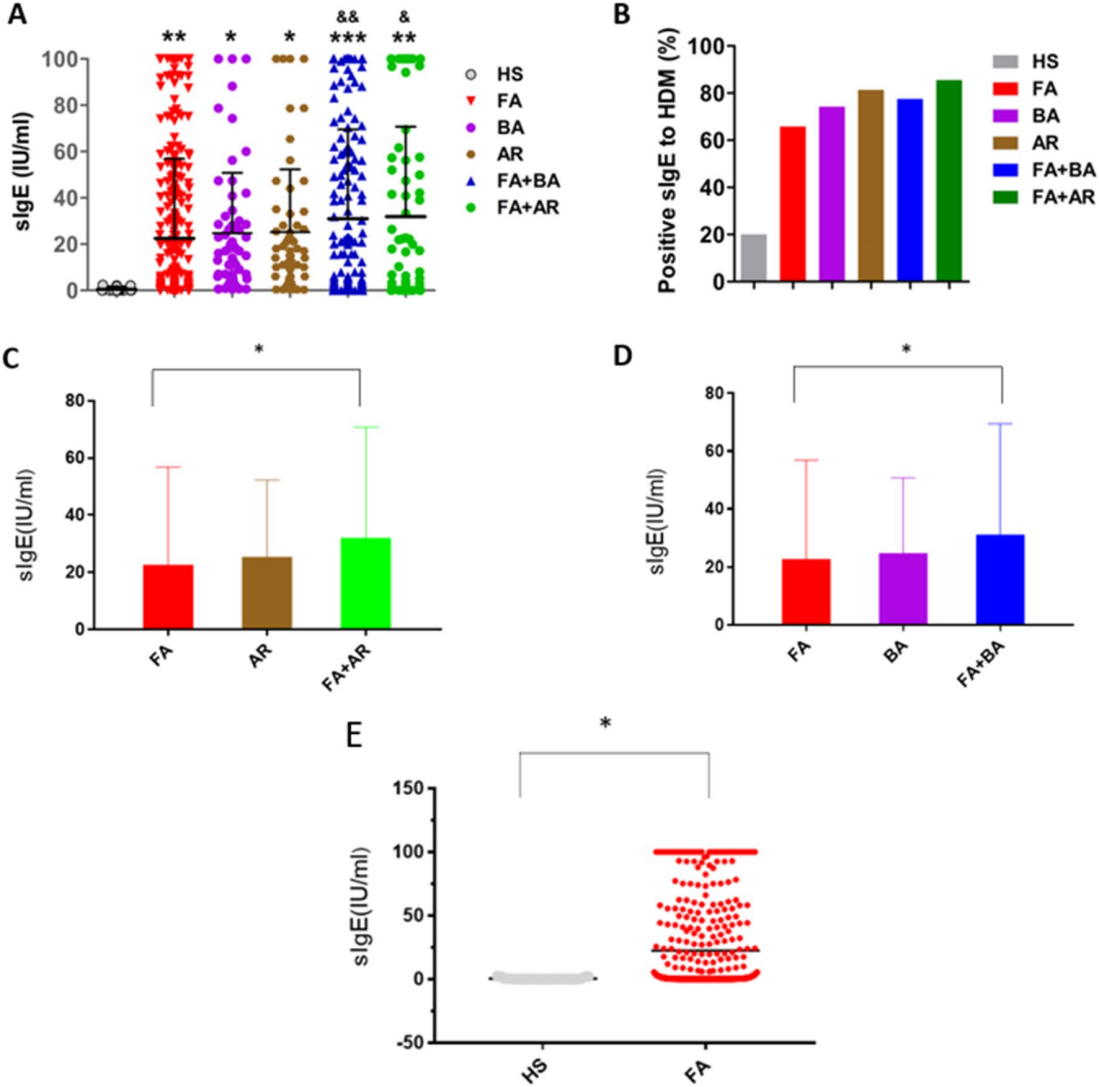
(**A**–**E**) All patients’ data were collected from the First Affiliated Hospital of Guangzhou Medical University. Including HS (healthy control) without allergic disease, 25 cases; FA (food allergy) without asthma, allergic dermatitis and allergic rhinitis, 403 cases; BA (bronchial asthma), 61 cases; AR (allergic rhinitis), 59 cases; FA + BA (food allergy with asthma), 223 cases; FA + AR (Food allergy with allergic rhinitis), 83 cases. (**A**) the levels of IgE against HDM. (**B**) the percentage of IgE against HDM. Those with HDM-sIgE level > 0.35 IU/ml were regarded as IgE positive. (**C**–**E**) the levels of IgE against HDM. FA + AR (Food allergy with allergic rhinitis), FA + BA (food allergy with asthma), FA (food allergy) without asthma, allergic dermatitis and allergic rhinitis. *p < 0.05, **p < 0.01, ***p < 0.001, ****p < 0.001, (ANOVA) compared with the HS group, and & *p* < 0.05, &&*p* < 0.01 compared with the FA group.

We collected 825 patients at our allergy clinic. As shown by Fig. 1A, compared to the healthy controls, the patients with food allergy have higher levels of HDM-specific IgE. Compared to the food allergy alone group (FA), the levels of specific IgE against HDM in the food allergy with allergic asthma group (FA + BA) or allergic rhinitis group (FA + AR) were increased significantly, while there was no significant difference between FA and allergic asthma alone group (BA) or allergic rhinitis alone group (AR). Those with HDM-sIgE levels > 0.35 IU/ml were regarded as IgE positive. About 20% healthy control subjects(HS) showed positive IgE reactivity againt HDM , while in FA, BA, AR, FA + BA and FA + AR, IgE reactivity with HDM were 65.9%, 74.1%, 84.35%, 77.5%, and 85.5%, respectively (Fig. 1B). Respectively, compared to AR or BA or HS, the levels of IgE against HDM were increased significantly in FA + AR (Fig. 1C) or FA + BA (Fig. 1D) or FA (Fig. 1E).

### Establishment of a mouse model of food allergy with HDM

**Figure 2:**
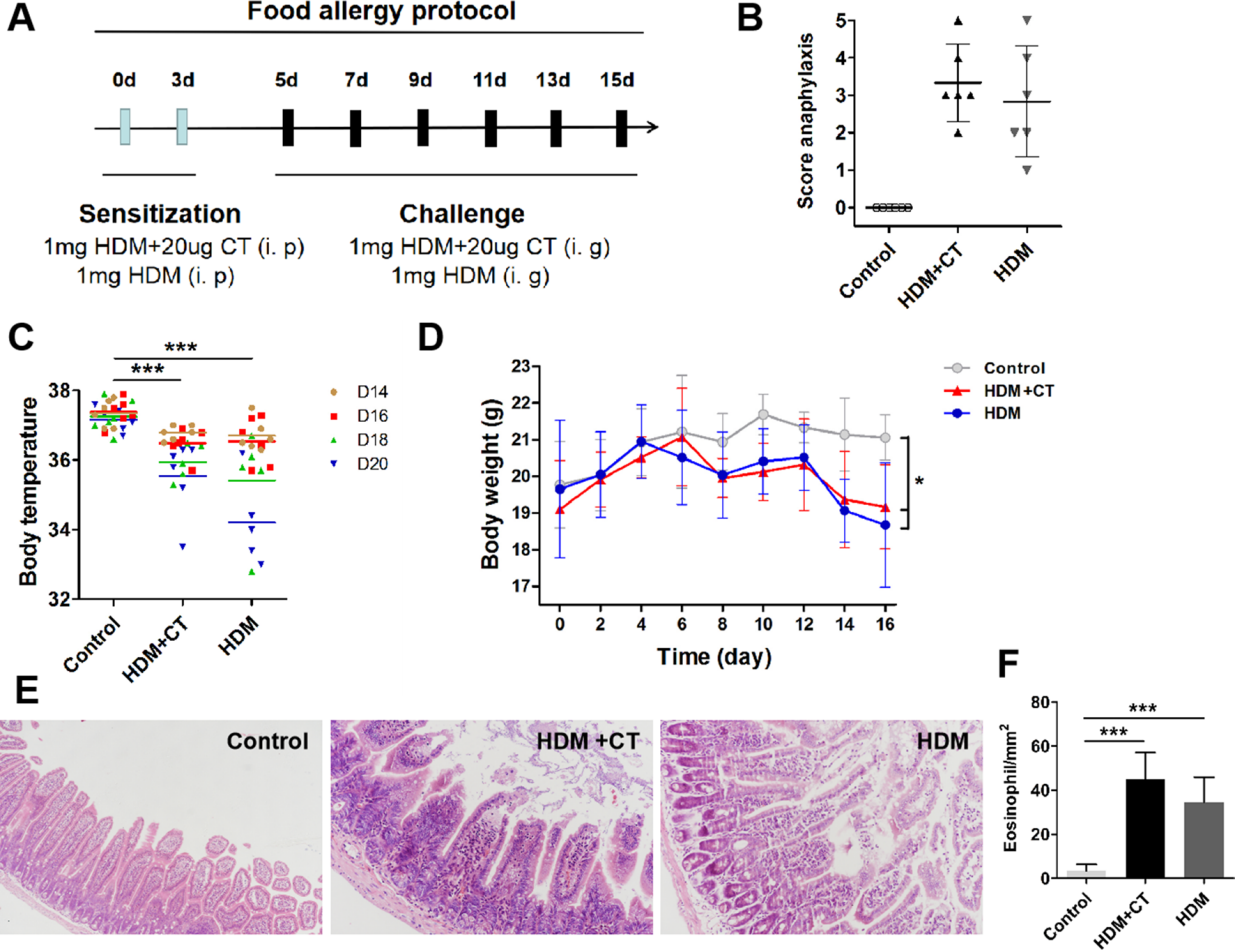
Establishment of a mouse model of food allergy with HDM. (**A**) The protocol for mouse model of HDM-induce food allergy. (**B**) the score of allergies of mouse. no symptoms, 0; scratch, scratch head and nose, 1; eye and mouth swelling, diarrhea, reduced activity and/or reduced activity with breathing emergency urge, 2; asthma, slow breathing, mouth and tail cyanosis, 3; After stimulate or shake, there is no activity, 4; death, 5. (**C**) the body temperature of mouse. (**D**) body weight of mouse. HE staining of jejunum. (**F**) the number of eosinophils in jejunum. Each group consists of 6 mice.*p < 0.05, **p < 0.01, ***p < 0.001, (ANOVA) compared to the control group. One mouse died in the HDM group on day 18.

As illustrated in Fig. 2A, mice were sensitized and challenged with HDM following the procedures we previously reported.^15^ After sensitization, the mice were challenged intragastrically with HDM. Systemic anaphylactic symptoms were evaluated within 30 to 40 min. All the mice in the HDM + CT group and the HDM group developed anaphylaxis (median anaphylactic score 3.3 and 2.83 respectively). Control mice showed no anaphylactic reactions (Fig. 2B). There was a decrease in body temperature during systemic anaphylaxis.^16^ Twenty-five minutes after HDM challenge, rectal temperature was measured. As shown in Fig. 2C, mice in HDM + CT group and HDM group showed significant reductions in rectal temperature than that of control group (P < 0.001). Allergic mice presented a metabolic change that leads to significant body weight loss compared with the control group.^17^ Consequently, HDM challenged mice presented body weight loss when compared with control mice (Fig. 2D). Furthermore, the contour of the jejunum from the control group mice were clear, and there was less inflammatory cell infiltration in the submucosa, whereas the inflamed jejunum of mice with HDM-treated revealed high levels of inflammatory cell infiltration, and sloughing of enterocytes at the tips of the villi (Fig. 2E–F).

### HDM facilitates Th2 immune response

**Figure 3:**
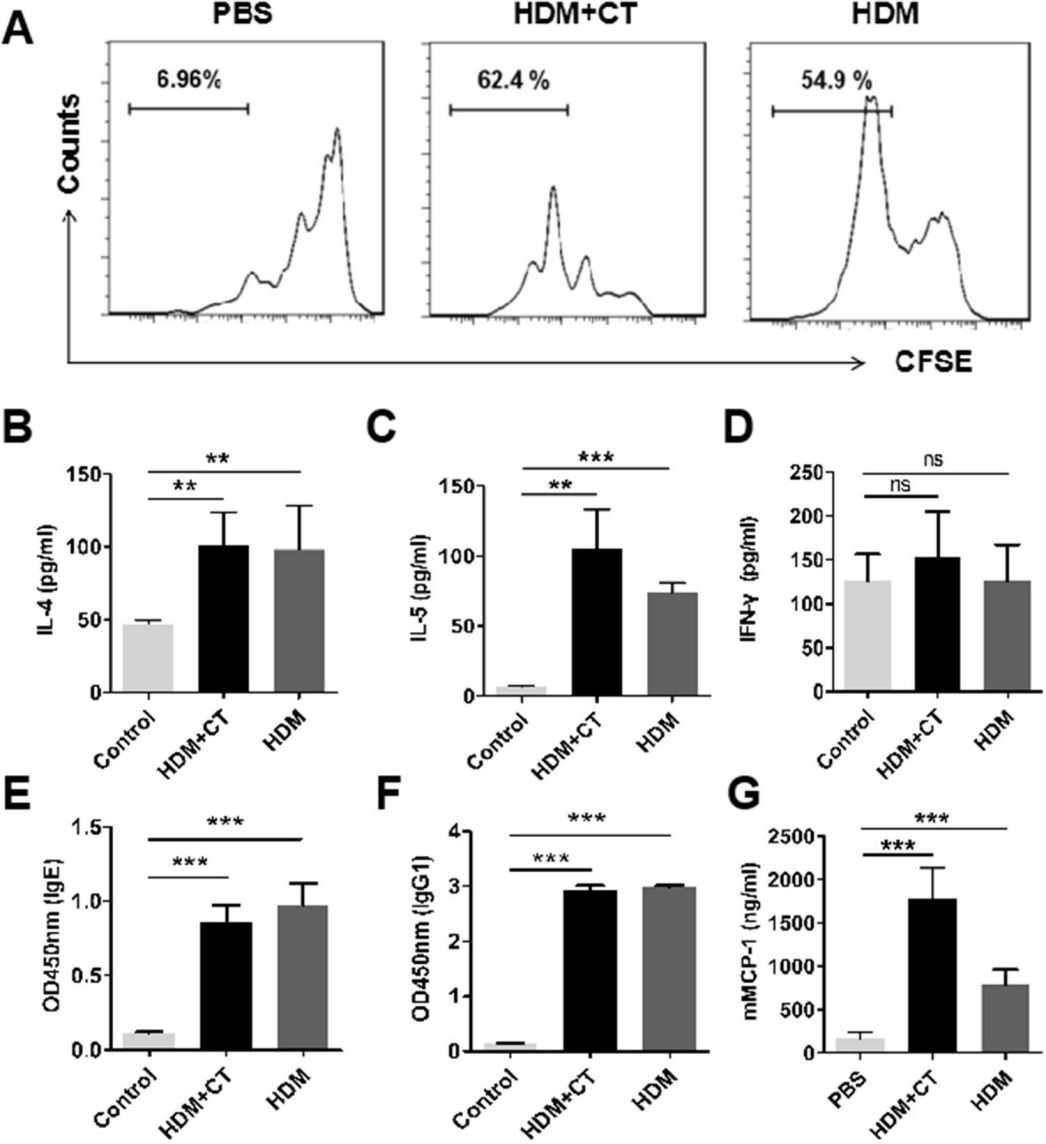
HDM facilitates Th2 immune response. (**A**) the histograms show the proliferation of CD4 + T cells. The bars show the levels of IL-4 (**B**), IL-5 (**C**) and IFN-γ (**D**) in the splenocyte culture supernatant. The level of serum HDM specific IgE (**E**), IgG1 (F) and mMCP-1 (G) were detected by EILSA. Each group consists of 6 mice, *p < 0.05, **p < 0.01, ***p < 0.001, (ANOVA) compared to the control group.

Spleen cells labeled with CFSE were cultured in the presence of HDM or saline for 72 h. The result showed that CD4 + CSFE-cells in the HDM + CT group and HDM group were more abundant than that in the control group, indicating that CD4^+^T cells markedly proliferated after stimulating with HDM (Fig. 3A). To further investigate whether HDM can enhance Th2 immune response, splenocyte cytokine profiles were analyzed in the present study. The result demonstrated that splenocytes from HDM-treated mice produced significantly high levels of Th2 cytokines (IL-4 and IL-5), but the levels of Th1 cytokine (IFN-γ) were not significantly changed (*P* > 0.05) (Fig. 3B–D). The specific immune response to HDM was also measured by testing the serum specific immunoglobulin levels. As shown in Fig. 3E–F, HDM-specific IgE and IgG1 levels were significantly increased in the HDM + CT group and HDM group. Mouse mast cell protease-1 (mMCP-1) is a marker of mast cell activation^18^. As shown in Fig. 3G, the mMCP-1 levels in the serum was higher in HDM-sensitized mice than that of the control group (*P* < 0.001).

### Exposure to HDM facilitates development of OVA-induced intestinal allergy

**Figure 4:**
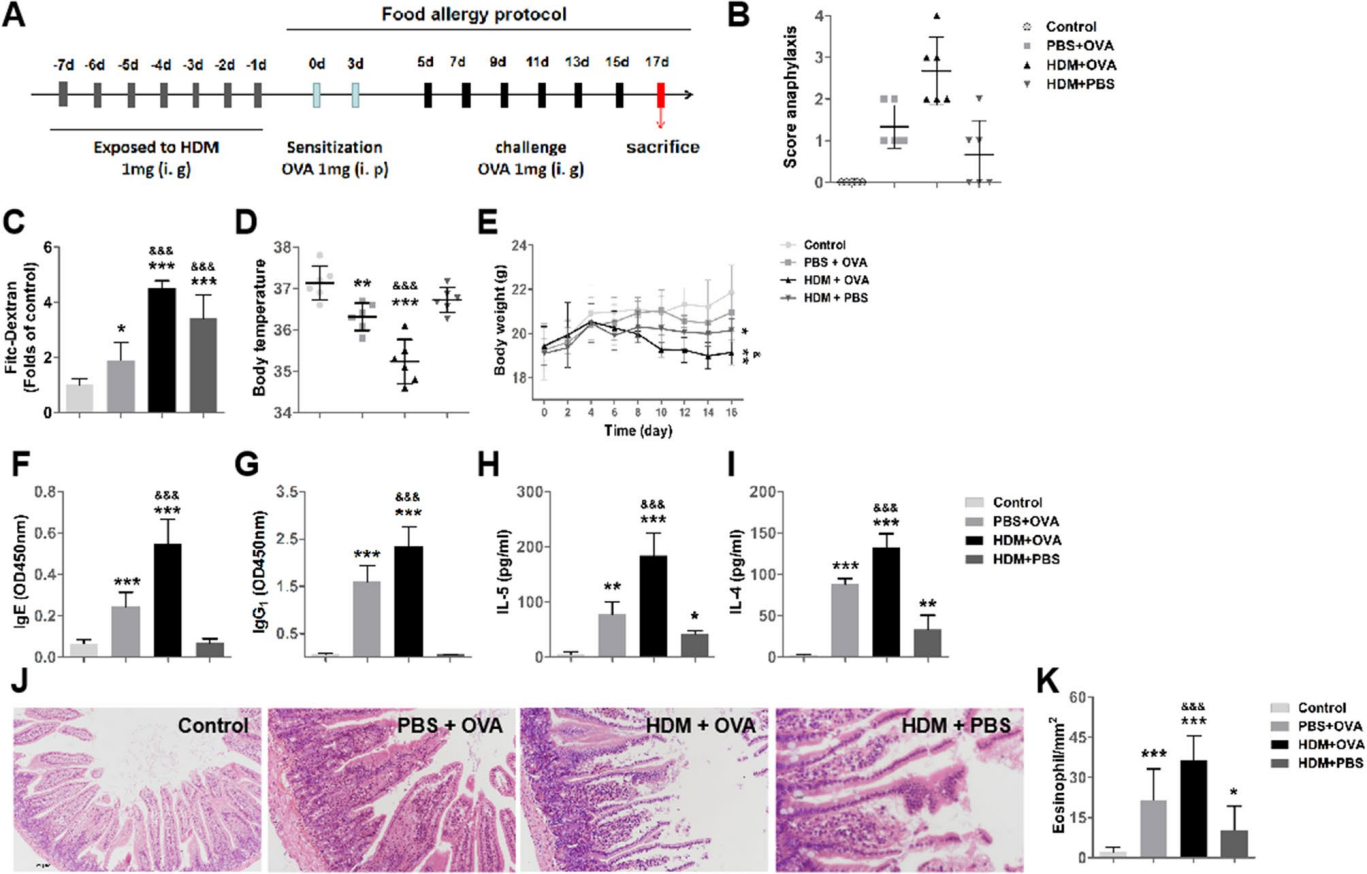
Exposure to HDM facilitates development of OVA-induced intestinal allergy. (**A**) The protocol for mouse model of HDM facilitates OVA-induce food allergy. (**B**) the score of allergies of mouse. no symptoms, 0; scratch, scratch head and nose, 1; eye and mouth swelling, diarrhea, reduced activity and/or reduced activity with breathing emergency urge, 2; asthma, slow breathing, mouth and tail cyanosis, 3; After stimulating or shaking, there is no activity, 4; death, 5. (**C**) Permeability to dextran (4,000-Da) of intestinal epithelial barrier in vivo. (**D**) the body temperature of mouse. (**E**) body weight of mouse. (**F**–**G**) The bars show the levels of serum OVA specific IgE and IgG1. (**H**–**I**) The bars show the levels of IL-4 and IL-5 in the splenocyte culture supernatant. (**J**) HE staining of jejunum. (**K**) the number of eosinophils in the jejunum. Each group consists of 6 mice. *p < 0.05, **p < 0.01, ***p < 0.001, (ANOVA) compared to the control group. &*p* < 0.05, &&*p* < 0.01, &&&*p* < 0.01 (*t* test) was compared to the PBS + OVA group.

HDM exposure aggravated the allergy-like symptoms, increases in permeability of intestinal epithelial barrier, lower body temperature, and lower body weight in OVA-induced intestinal allergy (Fig. 4B–E). The levels of sIgE, sIgG1, IL-4 and IL-5 in the HDM + OVA groups were higher in the HDM + OVA group than that the PBS + OVA groups and control groups (Fig. 4F–I). The pathological changes of the intestinal tract in the HDM + OVA group were more severe, and exhibited more eosinophils than PBS + OVA groups and control groups (Fig. 4J–K).

## Discussion

A large number of studies have been conducted to highlight the critical role of HDM allergen exposure, particularly in respiratory allergic diseases. In contrast, although HDM was known as an allergen, little attention was attracted in the study of food allergy. Tulic et al. reported recently that HDM allergen was detected in the human gastrointestinal tract, and the intestinal barrier function was affected directly by the cysteine protease activity of HDM allergen without prior sensitisation^10^. Nevertheless, the contribution of HDM in the pathogenesis of food allergy remains unknown. We carried out this study to elucidate that HDM is a crucial environmental trigger factor for developing food allergy.

Specific IgE antibodies play an important role in mediating type 1 allergic reaction in human^19^. Specific IgE that have already bound to the surface of mast cells or basophils can be bound by food allergens to cause the release of the allergy-related mediator such as histamines; subsequently, allergic symptoms occur. In this study, we observed that among 825 food-allergic patients, 65.9% exhibited IgE reactivity to HDM. Compared to FA patients, the levels of IgE against HDM in FA + BA or FA + AR were increased significantly, while there was no significant difference between FA and BA or AR. These data indicate that HDM may be an important contributor to food allergy.

A HDM-induced mouse model of food allergy was developed successfully in the present study, and hypersensitivity was evaluated with well-established parameters, including allergy-like symptoms scores, decreased body temperatures, heavy infiltration of inflammatory cells in the jejunal mucosa^16,20^, increased serum mMCP-1 levels^21^ and decreased body weight^22^, which demonstrate that HDM induces intestinal allergy in a mouse model.

Cytokines, secreted by T helper type 2 (Th2) cells, such as IL-4 and IL-5, are the major pathological feature of allergic disease, including food allergy.^22^ Especially, IL-4 promotes the production of allergen-specific IgE and activates mast cells to mediate type 1 inflammation in food allergy^23^. IL-5 plays an important role in the proliferation, recruitment and activation of eosinophils, and then promotes the development of type 1 inflammation^24^. In this study, we found that mice exposed to HDM showed significantly higher levels of Th2 cytokine, including IL-4 and IL-5, but not T helper type 1 (Th1) cytokine, IFN-γ, indicating that HDM facilitates Type 2 inflammation in mice.

CT is a potent mucosal adjuvant for stimulating allergen-specific immune response^25^, is also considered as a potent Th2 adjuvant because it stimulates production of Th2 cytokines and promotes specific IgE and IgG1 production^26^. In the present study, we fed mice with HDM (1 mg/mouse) in the presence of CT as an adjuvant. The group 1 allergen of *Dermatophagoides pteronyssinus* (Der p1) has been proven as the major HDM allergen, the proteolytic activity of Der p1 resulted in a significant reduction in IL-12 production in dendritic cells (DCs), and that DCs induced naive T cells (Th0) to secret less Th1 cytokine and more Th2 cytokine^27^. It is reported that T cell immunoglobulin mucin domain (TIM)4 expressed by antigen-presenting cells (APCs) that ligates TIM1 on Th2 cells, and the TIM-1-TIM-4 interaction promote Th2 cell polarization^28^. Mo LH et al. reported that exposure to Der p1 induce the TIM4 gene transcription and expression in DCs^29^, indicating that Der p1 can induce DCs to produce more TIM4, and induce Th2 polarization subsequently. Tulic et al. showed that Der p1 was present in the human intestine, the proteolytic activity of Der p1 resulted in disrupted of TJ proteins, reduced integrity of the mucus barrier, as well as increased permeability of epithelial cells^10^. Therefore, there is a hypothesis that some HDM proteins, such as Der p1, can act as mucosal adjuvant, which facilitate Th2 polarization, contribute to the intestinal barrier dysfunction, and increase the allergen transportation across the intestinal epithelial barrier. In this study, mice were sensitized and challenged without any adjuvant, and the result show that HDM alone induced intestinal allergy in mice. To further test our hypothesis, an OVA food allergy followed by HDM exposure were induced in mice. Consequently, results show that the exposure to HDM is able to induce intestinal barrier dysfunction, and facilitate the development of intestinal allergy in mice.

## Conclusion

In conclusion, our data demonstrate that HDM contribute to the development of food allergy.

## Data Availability

All data generated or analyzed during this study are included in this published article and its additional files.

## Abbreviations

HDMs: House dust mites
HDM: House dust mite
mMCP-1: Mouse mast cell protease-1
CT: Choleweighti n toxin
ELISA: Enzyme-linked immunosorbent assay
CBS: Carbonate buffered solution
BSA: Bovine serum albumin
CFSE: 5,6-Carboxyfluorescein diacetate, succinimidyl ester
PMA: Horbol 12-myristate 13-acetate
FITC: Fluorescein isothiocyanate
HE: Hematoxylin and eosin
Th: T helper type
APCs: Antigen-presenting cells
DCs: Dendritic cells
Der p1: The group 1 allergen of *Dermatophagoides pteronyssinus*
